# Risk of Cardiovascular Diseases and Cancer in Later Life: The Role of Age at First Marriage

**DOI:** 10.3390/geriatrics3020027

**Published:** 2018-06-07

**Authors:** Hui Liu, Zhenmei Zhang, Seung-won Choi

**Affiliations:** Department of Sociology, Michigan State University, Berkey Hall, 509 E. Circle Drive 316, East Lansing, MI 48824, USA; zhangz12@msu.edu (Z.Z.); choise23@msu.edu (S.-w.C.)

**Keywords:** age at first marriage, cardiovascular diseases, cancer, aging, gender

## Abstract

The objective of this study was to examine how age at first marriage is related to the risk of developing cardiovascular diseases and cancer in later life. We analyzed longitudinal data from a nationally representative sample of 2129 older adults (born in the 1940s or earlier) in the National Social Life, Health, and Aging Project. We found that for men in this cohort, the age at first marriage that was related to the lowest risk of cardiovascular diseases (CVD) and cancer in later life was the early 30s; men who first married at either younger or older ages had significantly higher odds of experiencing CVD and cancer. Interestingly, for women in this cohort, the age at first marriage was not related to the risk of either CVD or cancer.

## 1. Introduction

People are getting married later than ever before [1,2]. While this increase in age at first marriage is a well-documented demographic trend, the health consequences of delaying the age at first marriage are not fully understood. Research has found that, in general, delayed marriage has several advantages, particularly for college-educated women, in terms of socioeconomic achievement and marital stability [3,4]. On the other hand, there are also potential costs related to delaying marriage (e.g., difficulties in assortative mating in the marriage market) [5,6]. However, few studies have examined how the age at first marriage is related to the risk of development of chronic conditions in later life. Chronic diseases—such as cardiovascular diseases (CVD) and cancer—account for most deaths in the United States; and the risk of these conditions is much higher at older ages. Such age-dependent chronic diseases can severely damage quality of the life for the elderly and represent major barriers to healthy aging.

Previous studies on age at first marriage have focused primarily on the effects of early marriage on socioeconomic achievement, family stability, and mental health outcomes during young adulthood, suggesting that early first marriage, especially as a teenager, is related to worse outcomes during young adulthood [7,8]. Less attention has been paid to the long-term effects of age at first marriage on physical health at older ages. Among the limited empirical studies, one study using data from the 1992–2006 Health and Retirement Study (HRS) found that late marriages were associated with a lower mortality risk, especially for older men, but found no significant associations for older women [9]. There is also evidence showing that, especially for more highly educated women, late marriage may be associated with positive outcomes such as greater likelihood of marital success and higher socioeconomic achievement [5], which are likely to lead to better physical health and lower risk of chronic conditions in later life [10,11]. Yet, other studies have found no evidence that late age at first marriage is related to either of these life outcomes [3,4,12] or to chronic disease onset in later life [13]. 

In this study, we examine how age at first marriage modifies individual life context, which, in turn, affects the risk of developing CVD and cancer in later life. We analyze a nationally representative longitudinal dataset drawn from the first two waves of the National Social Life, Health, and Aging Project (NSHAP) to address two research questions: (1) Is age at first marriage related to the risk of developing CVD and cancer in later life? (2) Are there gender differences in these linkages? The importance of this study is highlighted by the continued very high prevalence of CVD and cancer among older adults in the United States as well as the severe consequences of these conditions on healthy aging and quality of life.

### 1.1. Data and Study Sample

We used data from the first two waves of the National Social Life, Health, and Aging Project (NSHAP), a nationally representative longitudinal dataset. The NSHAP is one of the first national-scale, population-based studies of health and intimate relationships. The survey was conducted by the National Opinion Research Center (NORC, Chicago, IL, USA) at the University of Chicago. A nationally representative probability sample of community-dwelling individuals aged 57–85 was selected from households across the United States in 2004. African Americans, Latinos, men, and those 75–84 years old at the time of screening were over-sampled. The NSHAP is uniquely suited for the purpose of this study because of the rich data it provides on marital and family transitions, socioeconomic factors as well as health conditions in later life. All analyses are weighted. We used the survey data analysis commands (SVY) in Stata [14] to account for the clustering and stratification in the complex sampling design.

The first wave of the NSHAP (Wave 1) included a sample of 3005 adults aged 57–85 who were interviewed in 2005–2006 [15]. In-home interviews were conducted as well as lab tests and assays. Wave 2 consisted of 2261 Wave 1 respondents who were re-interviewed in 2010–2011 [16]. Our analytic sample included only the respondents who were interviewed in both waves. Compared with those who were interviewed in both waves, those who were lost to follow-up at Wave 2 were more likely to report poorer health, more chronic conditions, be older, and to have lower levels of education and family income. This suggests that our sample may have excluded respondents who were relatively more disadvantaged—indicating a potentially more conservative finding. Moreover, our sensitivity analysis (i.e., applying the approach developed by Heckman [17]) to adjust the sample attrition biases suggested that our findings are robust with the correction for sample attrition due to loss to follow-up (See Liu and Waite [18] for similar applications). Given our interest in the age at first marriage, we further restricted our sample to 2129 respondents (1019 men and 1110 women) who were either currently married or previously married at Wave 1. A small number of respondents (*n* = 15) who reported marrying before age 14 were excluded due to the rare occurrence of marriage at such a young age. The results from additional analyses (not shown but available upon request) including these cases revealed no differences in the findings. In the final analyses, we also excluded a small number of respondents with missing values on the focal dependent variable in the specific model. Thus, the final sample size varied slightly across analyses of different dependent variables.

### 1.2. Measures

*Age at first marriage.* Age at first marriage was calculated by subtracting the year of birth from the year of the first marriage. We included both the linear and quadratic forms of age at first marriage in order to understand potential nonlinear patterns. In our additional analysis (not shown but available upon request), we also coded age at first marriage as a categorical variable with various cutoff points; the results were generally consistent with the patterns reported in the paper. Detailed frequencies of age at first marriage categories are shown in Table A1 in Appendix A.

*CVD and cancer.* We focused on CVD and cancer because they are the top 2 leading causes of death in the United States, and they are directly affected by social behavioral factors and become increasingly prevalent at older ages [19,20]. During the in-home interviews, all NSHAP respondents were asked whether they had ever been told by a medical doctor that they had had a heart attack, heart failure, or stroke. Respondents who reported any of these *CVD events* were coded as 1, and all others were coded as 0. Respondents were also asked whether they had ever been told by a medical doctor that they had *any type of cancer* (1 = yes, 0 = no).

*Control covariates.* We controlled three types of covariates which may be related to both age at first marriage and the risk of CVD and cancer: socioeconomic status (SES), family characteristics, and demographic covariates. We included two measures of SES: *education* (1 = some college or higher degree, 0 = others) and *family income* (i.e., self-assessment in comparison to average Americans: below average (reference), average, above average and missing reports). We considered a number of indicators of family characteristics and transitions including *current marital status* (1 = unmarried, 0 = married), *ever divorced* (1 = yes, 0 = no), *number of children* (0 (reference), 1–3, 4 or more, and missing), and *age at child’s first birth* (younger than 20 (reference), 20–24, 25–29, 30 and older, and missing). In terms of demographic covariates, we controlled for *age* (57–64 (reference), 65–74, and 75–85), *race/ethnicity* (non-Hispanic white (reference), non-Hispanic black, Hispanic, and other), *currently smoke* (1 = yes, 0 = no), *currently drink alcohol* (1 = yes, 0 = no), *physical exercise* (1 = exercise more than three times per week, 0 = others), and *body mass index* (BMI). BMI was measured as a categorical variable with five categories: normal or underweight (BMI < 25, reference), overweight (25 ≥ BMI < 30), obese (30 ≥ BMI < 40), morbidly obese (BMI ≥ 40), and missing (WHO Expert Committee 1995). We stratified all analyses by *gender.* All control covariates were measured in Wave 1.

### 1.3. Statistical Methods

We applied the lagged dependent variable approach to predict the risk of CVD and cancer, respectively, at Wave 2, using the age at first marriage reported at Wave 1 and by controlling Wave 1 risk of CVD and cancer. We ran four logistic regression models. In the baseline model, we examined the relationship between age at first marriage and CVD or cancer, controlling for basic demographic and health behavior-related covariates. The second model added SES covariates in addition to the controls in the baseline model to determine whether SES explains the relationship between age at first marriage and later health. The third model included family characteristics and transitions variables in addition to the controls in the baseline model to understand whether family characteristics and transitions account for the association. In the final full model, we added all covariates. Because the results showed no substantive differences in the patterns observed in the second and third models in comparison to the final full model, we only report results from the baseline models and the full models. Because the literature on marriage and health has found important and longstanding differences between men and women [18,20], we stratified all analyses by gender. Results from *t*-tests (not shown but available upon request) suggest that all key gender differences were statistically significant at the level of *p* < 0.001.

## 2. Results

Table 1 includes the weighted descriptive statistics for all analyzed variables for men and women. These results show that the average age at first marriage is older for men than for women (23.72 vs. 21.09). In Wave 2, on average, men showed a higher prevalence of CVD (22.02% vs. 14.40%) and cancer (29.23% vs. 20.97%) than women.

Table 2 and Table 3 show the estimated odds ratios of age at first marriage for predicting CVD and cancer for men and women, respectively. The significant effect of the quadratic term of age at first marriage in the baseline model of Table 2 suggests that, for men, there is a significant nonlinear relationship between age at first marriage and risk of CVD and cancer. Results from the full model in Table 2 suggest that the significant nonlinear relationship between age at first marriage and cancer remained unchanged after controlling for SES and family transition covariates. The nonlinear effect of age at first marriage on CVD was reduced to marginal significance when SES and family transition covariates were included. The results of the full model of Table 2 also suggest that men with an above average household income have lower odds of reporting CVD events (OR = 0.548, *p* < 0.05) than men with below average household income. Men who have their first child after age 30 have marginally significant lower odds (OR = 0.439, *p* < 0.1) of reporting CVD events in later life than men who have their first child before age 20.

**Figure 1:**
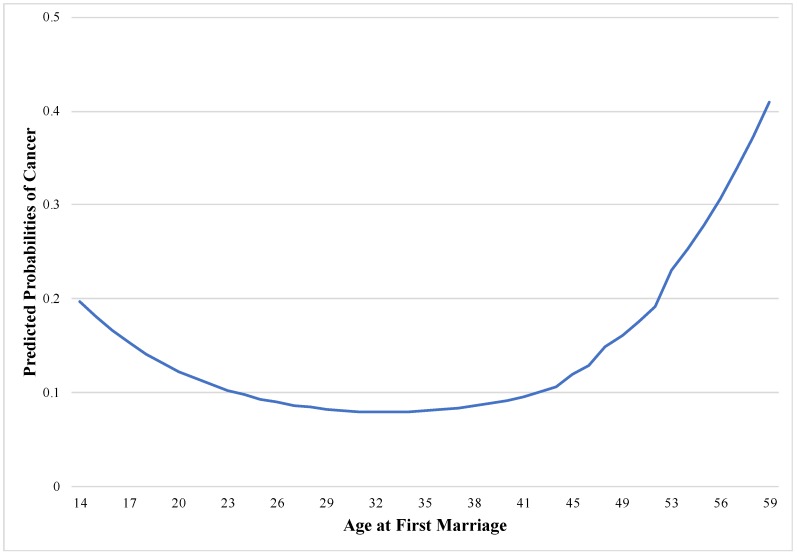
Cancer and age at first marriage for men.

To better illustrate the nonlinear patterns, we graphically present the results of cancer from the full model for men in Table 2. Figure 1 illustrates the relationship between age at first marriage and risk of cancer. We see that the odds of reporting cancer steadily decrease until the men’s age at first marriage reaches around the early 30s and then begin to increase with age at first marriage after the early 30s. Strikingly, the odds of reporting cancer were dramatically higher among men who first married after their 40s.

Interestingly, for women, as shown in Table 3, age at first marriage was not related to either CVD or cancer in all models. Additional analyses (results not shown but available upon request) that excluded the squared term of age at first marriage also revealed no significant linear relationship between age at first marriage and CVD/cancer for women.

## 3. Discussion and Conclusions

This study adds to a small but growing literature on the link between the timing of first marriage and physical health in later life [13,21,22]. We found a nonlinear relationship between age at first marriage and the risk of CVD and cancer among men but not women, holding constant the effects of age, race/ethnicity, health behaviors, and baseline health outcomes. Specifically, both early and—more strikingly—late first marriages were associated with a higher risk of CVD and cancer for men in later life (relative to first marriages between the mid 20s and the early 30s). This finding is consistent with several recent studies indicating that early first marriage, especially teenage marriage, is more detrimental to men’s physical health and survival than to women’s in both the United States [9,21,23] and other countries [22]. However, as far as we know, the current study is one of the first to find negative physical health outcomes related to very late first marriage (after age 30, and more dramatically, after age 40) for men. Our results suggest escalating risks of experiencing CVD and cancer for men who first marry after age 40. In addition, we found that after we controlled for SES and family characteristics and transitions, the relationship between age at first marriage and risk of cancer remained robust, but the association between age at first marriage and CVD risk was no longer statistically significant. This suggests that SES and family characteristics are potentially mediating variables that account for the negative effects of early and late marriage on men’s CVD risk. However, the etiology of cancer seems more complex, and none of our SES or family variables were associated with cancer risk. Future research needs to examine other factors, such as family history of cancer and occupation types as potential factors to explain the relationship between age at first marriage and cancer risk.

We speculate that men in our sample who got married at older ages might have faced strong social pressure to get married and their lives might have been quite stressful compared to men who married younger because their marital timing did not conform to social norms of the time. The stress of being “off-time” in entering marriage may have negative effects on men’s mental health [7] and could also lead to unhealthy behaviors, such as smoking and drinking, as coping strategies, all of which can increase the risk of CVD and cancer. It is also possible that the selection of healthier men into their first marriages in their late 20s (rather than either much younger or much older ages) may explain some of our findings. Moreover, it has long been argued that married men receive more health benefits from their marriage than married women do, because wives are more likely to provide emotional support and regulate health behaviors for their husband, whereas men are more likely to receive such benefits from their wife [24]. Men who marry at very late ages may not receive such health benefits from marriage, which may explain the finding of men’s poor health related to late age at marriage.

Surprisingly, we did not find any significant associations between the age at first marriage and the risk of CVD and cancer for women. We suspect that the lack of association between age at first marriage and late-life health conditions for women may be unique to this cohort of women in our sample (who were born in the 1940s or earlier). Due to their limited opportunities for pursuing higher education and careers in combination with a social norm of women getting married in their early twenties [4] may have meant that the effect of marital timing on education and labor force participation (thus on health) was not as significant as it is for women in more recent cohorts.

Although we were not able to provide direct evidence about the specific mechanisms underlying the association between marital timing and late-life disease development, this study makes a significant contribution to a growing body of literature on marital timing and chronic disease in later life. Using nationally representative longitudinal data from men and women in the United States, we found that both very early and very late first marriages were significantly associated with higher risks of CVD and cancer among older men, while marital timing was not associated with the risk of CVD or cancer among older women. We note that marital timing may have different social and economic consequences for the focal cohort than for more recent birth cohorts, and the findings may not be generalizable to younger cohorts. Given the high prevalence of chronic conditions among older adults, future research should continue to identify risk factors—not only downstream behavioral and biomedical factors but also upstream social factors associated with developing such conditions in order to promote healthy aging and preserve health-related quality of life for the elderly.

## Figures and Tables

**Table 1:** Weighted Descriptive Statistics of Analytic Variables.

Variables	All (*N* = 2129)	Men (*N* = 1019)	Women *(N* = 1110)	
	Mean (SD) (Range)			
Age at first marriage W1	22.35 (0.16)	23.72 (0.22)	21.09 (0.16)	*
	(14–64)	(14–64)	(14–61)	
	*N* (%)			
CVD W2 (yes = 1)	404 (18.05)	237 (22.02)	(14.40)	*
Cancer W2 (yes = 1)	561 (24.92)	315 (29.23)	(20.97)	*
*Control covariates W1*				
Some college or higher (yes = 1)	1305 (57.90)	669 (61.93)	(54.19)	*
*Relative household income*				
Below average (ref.)	564 (25.03)	223 (20.64)	340 (29.07)	*
Average	759 (33.67)	343 (31.75)	416 (35.44)	
Above average	554 (24.59)	326 (30.21)	228 (19.41)	*
Missing	377 (16.72)	188 (17.40)	189 (16.09)	
Currently unmarried (yes = 1)	717 (31.80)	191 (17.73)	525 (44.76)	*
Ever experienced divorce (yes = 1)	821 (36.44)	404 (37.39)	417 (35.56)	
*Number of children*				
No child (ref.)	121 (5.37)	56 (5.18)	65 (5.54)	
1–3	1219 (54.08)	574 (53.18)	644 (54.91)	
4 or more	528 (23.44)	242 (22.37)	286 (24.41)	
Missing	386 (17.12)	208 (19.26)	178 (15.14)	*
*Age at first birth*				
Before 20 (ref.)	386 (16.33)	73 (6.75)	295 (25.16)	*
20–24	745 (33.08)	326 (30.16)	420 (35.76)	*
25–29	428 (18.98)	265 (24.51)	163 (13.88)	*
30 or more	174 (7.70)	122 (11.27)	52 (4.41)	*
Missing	539 (23.91)	295 (27.30)	244 (20.79)	*
Female (=1)	1173 (52.11)			
*Age groups*				
57–64 (Ref.)	997 (44.23)	532 (49.24)	465 (39.73)	*
65–74	771 (34.23)	347 (32.17)	424 (36.19)	
75–85	485 (21.54)	201 (18.58)	284 (24.08)	*
*Race/ethnicity*				
Non-Hispanic white (ref.)	1826 (81.13)	874 (81.03)	953 (81.23)	
Non-Hispanic black	217 (9.62)	98 (9.07)	119 (10.12)	*
Hispanic	154 (6.84)	78 (7.21)	76 (6.50)	
Other races	54 (2.40)	29 (2.68)	25 (2.15)	
Smoking (yes = 1)	330 (14.66)	178 (16.48)	152 (12.98)	*
Drinking (yes = 1)	1345 (59.69)	736 (68.22)	609 (51.82)	*
Physical activity (≥3 times per week = 1)	1489 (66.17)	762 (70.55)	727 (62.12)	*
*BMI*				
Underweight or normal (ref.)	506 (22.50)	209 (19.40)	297 (25.37)	*
Overweight	798 (35.39)	210 (37.96)	387 (33.01)	*
Obesity	723 (32.07)	369 (34.14)	354 (30.17)	
Morbidly obese	106 (4.69)	37 (3.43)	69 (5.86)	*
Missing	122 (5.35)	55 (5.08)	66 (5.60)	
CVD W1 (yes = 1)	383 (17.05)	228 (21.18)	155 (13.25)	*
Cancer W1 (yes = 1)	541 (24.02)	272 (25.18)	269 (22.96)	

Note: BMI = Body Mass Index; W1 = Wave 1; W2 = Wave 2. * *p* < 0.05 between the results of men and women. The significance tests were based on two-tailed *t*-tests for continuous variables and two-tailed tests of proportions for categorical variables using Stata [14]. The test of proportion was a hypothesis test on the equality of proportions of two samples. Specifically, it compared the specific proportions between the male and female subsamples.

**Table 2:** Estimated Odds Ratios of CVD and Cancer by Age at First Marriage, Men.

Variables	CVD (*N* = 1005)		Cancer (*N* = 1012)	
	Baseline Model	Full Model	Baseline Model	Full Model
Age at first marriage	0.835 *	0.898	0.835 *	0.822 *
	(0.065)	(0.063)	(0.074)	(0.079)
Age at first marriage, squared	1.003 *	1.002 ^+^	1.003 *	1.003 *
	(0.001)	(0.001)	(0.001)	(0.001)
*Socioeconomic status*				
College or higher (yes = 1)		1.155		0.991
		(0.265)		(0.188)
Relative household income (ref: below average)			
Average		0.734		1.296
		(0.209)		(0.389)
Above average		0.548 *		1.452
		(0.142)		(0.326)
Missing		0.525 ^+^		0.884
		(0.183)		(0.257)
*Family characteristics and transitions*				
Currently unmarried (yes = 1)		0.669		1.271
		(0.187)		(0.335)
Ever experienced divorce (yes = 1)		0.977		0.977
		(0.150)		(0.167)
Number of children (ref: no child)				
1–3		0.644		0.858
		(0.327)		(0.424)
4 or more		0.697		1.067
		(0.320)		(0.659)
Missing		0.992		1.079
		(0.493)		(0.347)
Age at first birth (ref: younger than 20)				
20–24		0.732		1.232
		(0.250)		(0.546)
25–29		0.584		0.913
		(0.244)		(0.409)
30 or older		0.439 ^+^		1.492
		(0.203)		(0.803)
Missing		0.387 *		1.767
		(0.180)		(0.931)
*Other Covariates*				
Age groups (ref: 57–64)				
65–74	2.017 ***	1.939 **	2.448 ***	2.376 ***
	(0.374)	(0.370)	(0.484)	(0.454)
75–85	1.935	1.991 ^+^	1.725 *	1.669 *
	(0.794)	(0.795)	(0.427)	(0.424)
Race/ethnicity (ref: non-Hispanic white)				
Non-Hispanic black	0.755	0.802	0.798	0.683
	(0.189)	(0.187)	(0.298)	(0.269)
Hispanic	0.402 *	0.407 *	0.545 ^+^	0.547 ^+^
	(0.142)	(0.147)	(0.175)	(0.188)
Other	0.795	0.960	0.992	0.874
	(0.615)	(0.702)	(0.568)	(0.559)
Smoking (yes = 1)	1.870 *	1.833 *	0.820	0.858
	(0.500)	(0.439)	(0.209)	(0.209)
Drinking (yes = 1)	1.108	1.178	0.943	0.934
	(0.287)	(0.282)	(0.190)	(0.188)
Physical activity (≥3 times per week = 1)	0.866	0.859	0.645 *	0.648 *
	(0.206)	(0.186)	(0.109)	(0.118)
BMI (ref: underweight or normal)				
Overweight	1.668	1.761	1.136	1.124
	(0.567)	(0.628)	(0.282)	(0.284)
Obesity	1.914 *	1.932 *	0.736	0.731
	(0.607)	(0.584)	(0.195)	(0.191)
Morbidly obese	4.071 *	4.243 *	1.096	1.058
	(2.261)	(2.414)	(0.586)	(0.560)
Missing	1.433	1.418	1.426	1.187
	(0.707)	(0.720)	(0.696)	(0.579)
CVD W1	14.133 ***	1.761		
	(3.346)	(0.628)		
Cancer W1			12.864 ***	13.604 ***
			(3.421)	(3.788)

Note: Robust standard errors in parentheses. Two-tailed *t*-tests for individual coefficients: * *p* < 0.05; ** *p* < 0.01; *** *p* < 0.001; ^+^ *p* < 0.1.

**Table 3:** Estimated Odds Ratios of CVD and Cancer by Age at First Marriage, Women.

Variables	CVD (*N* = 1089)		Cancer (*N* = 1105)	
	Baseline Model	Full Model	Baseline Model	Full Model
Age at first marriage	1.006	1.140	1.073	1.143 ^+^
	(0.092)	(0.103)	(0.079)	(0.089)
Age at first marriage, squared	1.000	0.998	0.998	0.997 ^+^
	(0.001)	(0.002)	(0.001)	(0.001)
*Socioeconomic status*				
College or higher (yes = 1)		0.555 *		1.168
		(0.147)		(0.303)
Relative household income (ref: below average)			
Average		0.538 ^+^		1.034
		(0.167)		(0.218)
Above average		0.582		0.837
		(0.213)		(0.257)
Missing		1.124		1.112
		(0.401)		(0.385)
*Family characteristics and transitions*				
Currently unmarried (yes = 1)		0.778		0.759
		(0.198)		(0.192)
Ever experienced divorce (yes = 1)		1.173		1.202
		(0.286)		(0.265)
Number of children (ref: no child)				
1–3		3.441		0.374 ^+^
		(2.842)		(0.210)
4 or more		2.397		0.299 ^+^
		(2.083)		(0.193)
Missing		5.068 **		0.417 ^+^
		(2.751)		(0.205)
Age at first birth (ref: younger than 20)				
20–24		0.642		1.070
		(0.210)		(0.305)
25–29		0.753		0.535
		(0.394)		(0.232)
30 or older		0.373		1.060
		(0.308)		(0.503)
Missing		0.893		0.519
		(0.778)		(0.356)
*Other Covariates*				
Age groups (ref: 57–64)				
65–74	1.717 *	1.827 *	1.339	1.473
	(0.381)	(0.453)	(0.299)	(0.358)
75–85	2.607 ***	2.457 **	1.796 *	2.269 **
	(0.676)	(0.728)	(0.417)	(0.647)
Race/ethnicity (ref: non-Hispanic white)				
Non-Hispanic black	0.792	0.547 *	0.380 ***	0.426 ***
	(0.238)	(0.164)	(0.094)	(0.102)
Hispanic	0.304 **	0.197 ***	0.642	0.718
	(0.118)	(0.089)	(0.212)	(0.262)
Other	0.071 ^+^	0.056 ^+^	0.978	0.977
	(0.095)	(0.087)	(0.713)	(0.788)
Smoking (yes = 1)	0.404 *	0.327 *	1.385	1.389
	(0.178)	(0.153)	(0.504)	(0.522)
Drinking (yes = 1)	0.787	0.978	1.086	1.068
	(0.212)	(0.253)	(0.234)	(0.251)
Physical activity (≥3 times per week = 1)	0.893	1.018	0.743	0.720
	(0.248)	(0.276)	(0.148)	(0.143)
BMI (ref: underweight or normal)				
Overweight	0.655	0.589 ^+^	0.832	0.917
	(0.212)	(0.160)	(0.207)	(0.226)
Obesity	1.500	1.485	0.892	0.906
	(0.430)	(0.459)	(0.213)	(0.209)
Morbidly obese	0.788	0.845	0.528	0.519
	(0.270)	(0.315)	(0.269)	(0.264)
Missing	1.208	0.805	0.966	1.091
	(0.544)	(0.327)	(0.450)	(0.491)
CVD W1	21.290 ***	26.375 ***		
	(4.863)	(7.335)		
Cancer W1			8.092 ***	8.435 ***
			(1.605)	(1.772)

Note: Robust standard errors in parentheses. Two-tailed *t*-tests for individual coefficients: * *p* < 0.05; ** *p* < 0.01; *** *p* < 0.001; ^+^ *p* < 0.1.

## Appendix A

**Table A1:** Frequencies of Age at First Marriage Categories for Analytic Samples, *N* (%).

	Analytic Sample for CVD			Analytic Sample for Cancer		
Age at First Marriage	All (*N* = 2094)	Men (*N* = 1005)	Women (*N* = 1089)	All (*N* = 2117)	Men (*N* = 1012)	Women (*N* = 1105)
≤19	576 (27.51)	150 (14.93)	426 (39.12)	586 (27.68)	151 (14.92)	435 (39.37)
20–29	1330 (63.51)	729 (72.54)	601 (55.19)	1341 (63.34)	734 (72.53)	607 (54.93)
≥30	188 (8.98)	126 (12.54)	62 (5.69)	190 (8.97)	127 (12.55)	63 (5.70)
